# Genetic evidence for the causal association between type 1 diabetes and the risk of polycystic ovary syndrome

**DOI:** 10.1186/s40246-023-00550-z

**Published:** 2023-11-13

**Authors:** Shuwen Chen, Zaixin Guo, Qi Yu

**Affiliations:** Department of Obstetrics and Gynecology, National Clinical Research Center for Obstetric and Gynecologic Diseases, State Key Laboratory of Complex Severe and Rare Diseases, Peking Union Medical College Hospital, Chinese Academy of Medical Sciences and Peking Union Medical College, Peking Union Medical College Hospital (Dongdan Campus), No.1 Shuaifuyuan Wangfujing Dongcheng District, Beijing, 100730 China

**Keywords:** Type 1 diabetes (T1D), Polycystic ovary syndrome (PCOS), Mendelian randomization (MR), Causal effect, Genetic epidemiology

## Abstract

**Background:**

Accumulating observational studies have identified associations between type 1 diabetes (T1D) and polycystic ovary syndrome (PCOS). Still, the evidence about the causal effect of this association is uncertain.

**Methods:**

We performed a two-sample Mendelian randomization (MR) analysis to test for the causal association between T1D and PCOS using data from a large-scale biopsy-confirmed genome-wide association study (GWAS) in European ancestries. We innovatively divided T1D into nine subgroups to be analyzed separately, including: type1 diabetes wide definition, type1 diabetes early onset, type 1 diabetes with coma, type 1 diabetes with ketoacidosis, type 1 diabetes with neurological complications, type 1 diabetes with ophthalmic complications, type 1 diabetes with peripheral circulatory complications, type 1 diabetes with renal complications, and type 1 diabetes with other specified/multiple/unspecified complications. GWAS data for PCOS were obtained from a large-scale GWAS (10,074 cases and 103,164 controls) for primary analysis and the IEU consortium for replication and meta-analysis. Sensitivity analyses were conducted to evaluate heterogeneity and pleiotropy.

**Results:**

Following rigorous instrument selection steps, the number of SNPs finally used for T1D nine subgroups varying from 6 to 36 was retained in MR estimation. However, we did not observe evidence of causal association between type 1 diabetes nine subgroups and PCOS using the IVW analysis, MR-Egger regression, and weighted median approaches, and all *P* values were > 0.05 with ORs near 1. Subsequent replicates and meta-analyses also yielded consistent results. A number of sensitivity analyses also did not reveal heterogeneity and pleiotropy, including Cochran’s *Q* test, MR-Egger intercept test, MR-PRESSO global test, leave-one-out analysis, and funnel plot analysis.

**Conclusion:**

This is the first MR study to investigate the causal relationship between type 1 diabetes and PCOS. Our findings failed to find substantial causal effect of type 1 diabetes on risk of PCOS. Further randomized controlled studies and MR studies are necessary.

**Supplementary Information:**

The online version contains supplementary material available at 10.1186/s40246-023-00550-z.

## Background

Polycystic ovary syndrome (PCOS) is probably the most common reproductive endocrine disorder among women of childbearing age, with a global prevalence of about 6–15% in the general population, and is characterized by chronic ovulation disorders, hyperandrogenemia and polycystic ovarian morphology (PCOM) [1–3]. This heterogeneous syndrome may have a significant impact on metabolic homeostasis of various body systems, reproductive function and psycho-emotional consequences, and therefore, early recognition and adequate appropriate management are essential for these women to improve their quality of life and reduce future long-term complications [4]. There are three major international diagnostic criteria for adult PCOS, including the diagnostic consensus developed by the National Institutes of Health (NIH) in 1990 [5], the diagnostic criteria further expanded by the European Society of Human Reproduction and Embryology (ESHRE) and the American Society for Reproductive Medicine (ASRM) in Rotterdam in 2004 [6], and the diagnostic criteria for PCOS developed by the Androgen Excess and PCOS Society (AE-PCOS) in 2006 [7]. The combination of these diagnostic criteria varies depending on the definition used. Thus, it follows that PCOS is a highly heterogeneous clinical syndrome. Moreover, due to the similarities between some physiological changes in puberty and the clinical manifestations of PCOS, no clear diagnostic criteria have been formulated for pubertal PCOS, and the corresponding diagnostic techniques are extremely limited.

Type 1 diabetes (T1D) is an autoimmune disease that causes pancreatic β-cell damage, leading to a decrease in insulin synthesis or a complete lack of insulin secretion in patients, which results in elevated blood glucose [8]. Although most patients with T1D do not have generalized and abdominal obesity, some studies have found adipocyte insulin resistance in children and adults with T1D [9, 10]. Adipose tissue also secretes a number of factors that not only regulate adipocyte, macrophage and multifunctional cell function, but also affect ovarian function, either directly or indirectly on granulosa cells (e.g., interleukin 6, leptin, and adiponectin), or on cumulus cells and oocytes (e.g., leptin and adiponectin) [11, 12]. Recent studies have found that reproductive dysfunction is common in women with type 1 diabetes, and up to 40% of women may have varying degrees of menstrual or reproductive disorders throughout their life [13], including delayed puberty and primary amenorrhea, low fertility, and the well-known complications of pregnancy and early menopause [13].

There are also studies suggesting a possible association between T1D and PCOS; unfortunately, the interaction and pathogenesis of T1D and PCOS remain unclear [14, 15]. Insulin replacement therapy has been widely used in clinical T1D patients to tightly control the metabolic status and prevent the development of long-term complications associated with persistent hyperglycemia [16]. Although there are structural similarities between exogenous and endogenous insulin, the subcutaneous administration of insulin replacement therapy is non-physiologic, and thus, a supraphysiologic dose of insulin needs to be injected subcutaneously in order to achieve the physiologically required concentration in the portal vein to inhibit blood glucose synthesis in the liver. This leads to an inevitable therapeutic increase in the dose of insulin therapy, resulting in exogenous systemic hyperinsulinemia, which stimulates androgen over-synthesis and secretion in the ovaries and adrenal glands, which in turn may lead to the development of PCOS [16]. However, comparing different insulin treatment modalities did not reveal differences in the prevalence of PCOS and its symptoms [17]. In addition, comparisons between well-controlled and poorly controlled adolescents with T1D did not reveal differences in androgen concentrations and ovarian parameters [17]. It is also worth noting that the biochemical and clinical manifestations of type 1 diabetes combined with PCOS may differ from those of women with PCOS alone. Women with type 1 diabetes combined with PCOS may have normal or elevated levels of SHBG and lower concentrations of free androgens [18]. Regarding the association between PCOS and T1D, there are no randomized clinical trials (RCTs), although there have been basic experimental studies and clinical observational studies [14, 17, 18]. Also, many of them are retrospective studies, and the temporal relationship of disease occurrence cannot be determined. Not only that, many studies have a large number of confounding factors, with a high degree of heterogeneity and a lack of causality inference. Due to the profound impact of PCOS on women's reproductive function and body metabolism, it is crucial to screen for the prevalence of PCOS in women with type 1 diabetes in reproductive age.

Mendelian randomization (MR) is an observational methodology whereby genetic variants are used as instrumental variables (IVs) to estimate the causal effect of an exposure (in this case, type 1 diabetes) on an outcome (development of PCOS) [19, 20]. Since the IVs analysis mimics RCT with respect to the random allocation of single nucleotide polymorphisms (SNPs) at conception prior to disease onset (independent of confounding factors such as sex and age), MR is less susceptible to reverse causality, confounding than other observational studies and identify causal determinants of a certain outcome [20]. In addition, RCTs on this topic are impractical due to the requirement of significant personnel resources and time-consuming follow-up. In the absence of RCTs, MR is increasingly being used to infer credible causal relationships between risk factors and disease outcomes [20].

Given the uncertainty about the causal association between type 1 diabetes and PCOS, we conducted two-sample MR to investigate the potential causal effect using large-scale genome-wide association study (GWAS) data. And we also considered the time of onset of type 1 diabetes (whether it was early or not) and different comorbidities divided into nine subgroups for MR analysis. Overall, this study assesses the impacts of T1D on PCOS and provides constructive suggestions for preventive intervention strategies.

## Results

### Study overview

**Fig. 1:**
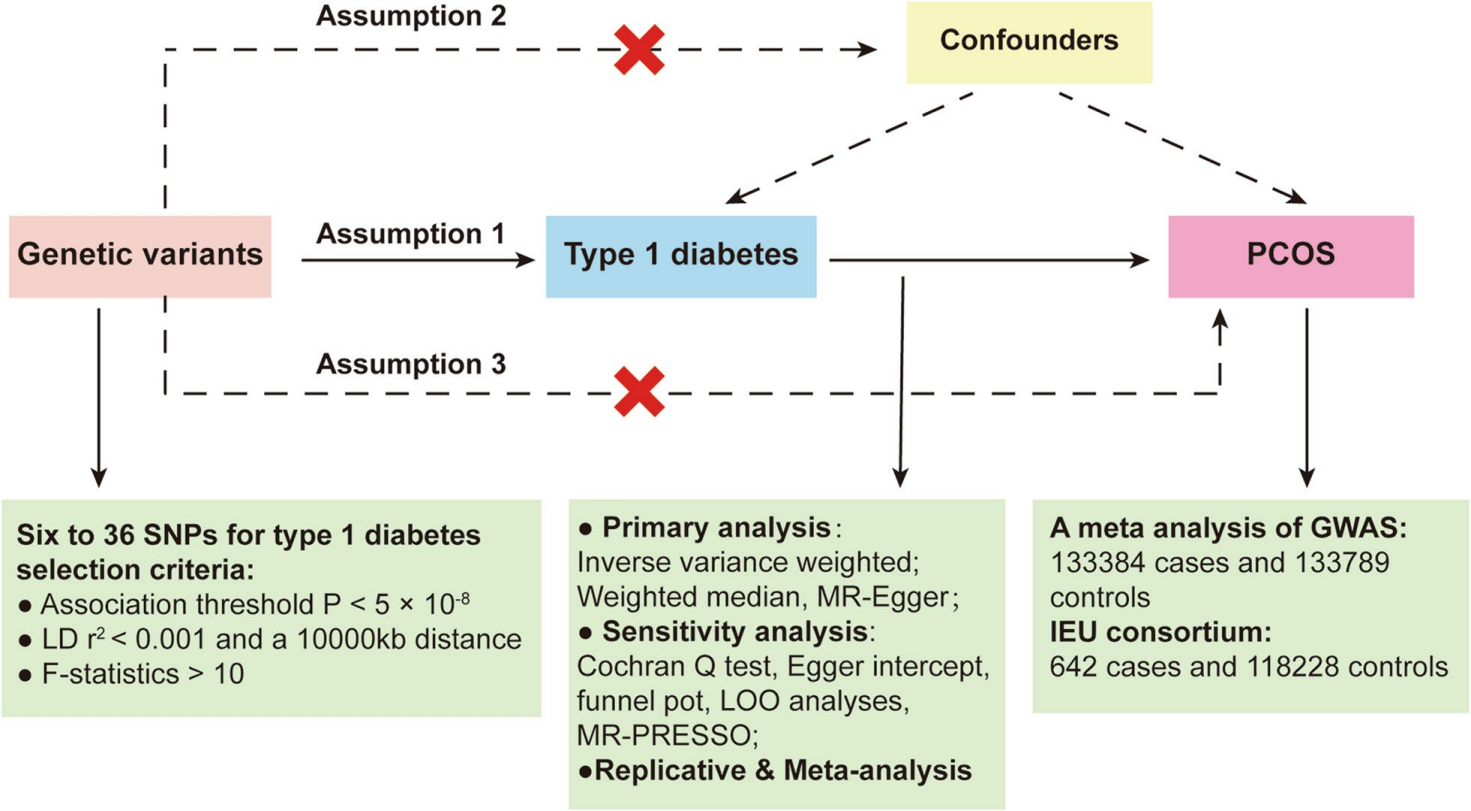
Workflow of the current Mendelian randomization (MR) study revealing causality from type 1 diabetes on PCOS. Assumption 1, genetic variants are robustly associated with exposure; Assumption 2, genetic variants are not associated with confounders; Assumption 3, genetic variants affect the outcomes only through the exposure of interest. SNPs, single nucleotide polymorphisms; LD, linkage disequilibrium; LOO, leave-one-out; MR-PRESSO, MR Pleiotropy RESidual Sum and Outlier

The underlying assumptions of two-sample Mendelian randomization are that instrumental variables are strongly correlated with exposure (assumption 1, the association hypothesis) and that instrumental variables cannot be directly correlated with the outcome variable, but can only influence the outcome through exposure factors (assumption 3, the exclusivity hypothesis). As shown in Fig. 1, we selected SNPs significantly associated with exposure (T1D) at the genome-wide significance levels (*P* < 5 × 10^−8^) according to rigorous instrument selection steps written in the Methods section. At the same time, SNPs with linkage disequilibrium (*R*^2^ threshold = 0.001 and window size = 10 mb) were aggregated and the F-statistic was calculated to measure statistical strength. All of the above methods ensure that the genetic markers we screened were strongly associated with our exposure (T1D) and may better measure the causal relationship with outcome (PCOS).

And the number of SNPs finally used for type 1 diabetes nine subgroups varying from 6 to 36 was retained in MR estimation (Additional file 2: Tables S3–S11). F statistics for SNPs were ranged from 128.07 to 419.62, all over 10, suggesting the strong genetic instruments were employed (Additional file 2: Tables S3–S11). The harmonized data are presented in Additional file 2: Tables S3–S11.

### MR estimates

**Fig. 2:**
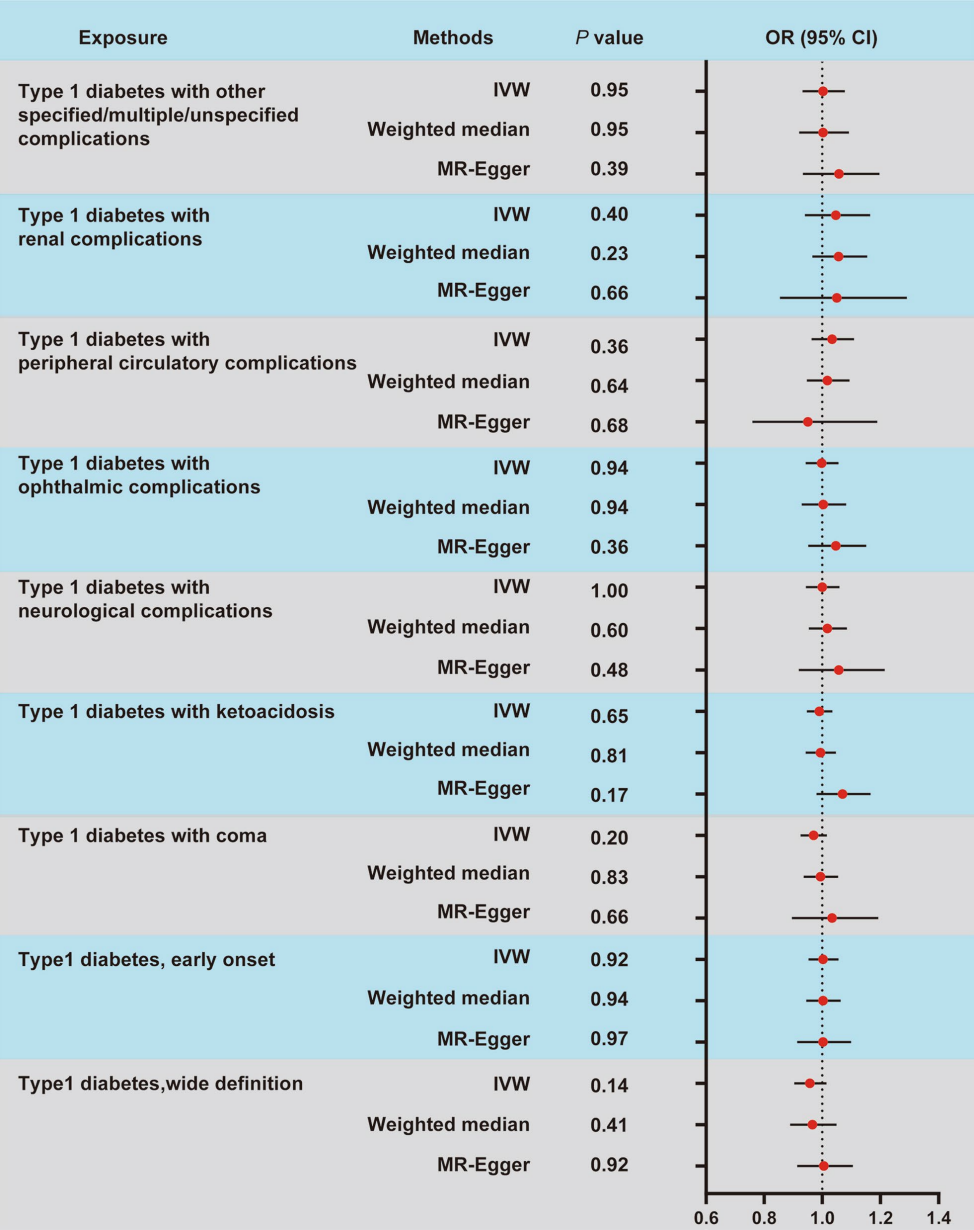
Forest plot for the causal effect of type 1 diabetes on the risk of PCOS. Summary of the MR estimates derived from the inverse-variance weighted (IVW), weighted median (WM), and MR-Egger. OR, odds ratio; CI, confidence interval

**Fig. 3:**
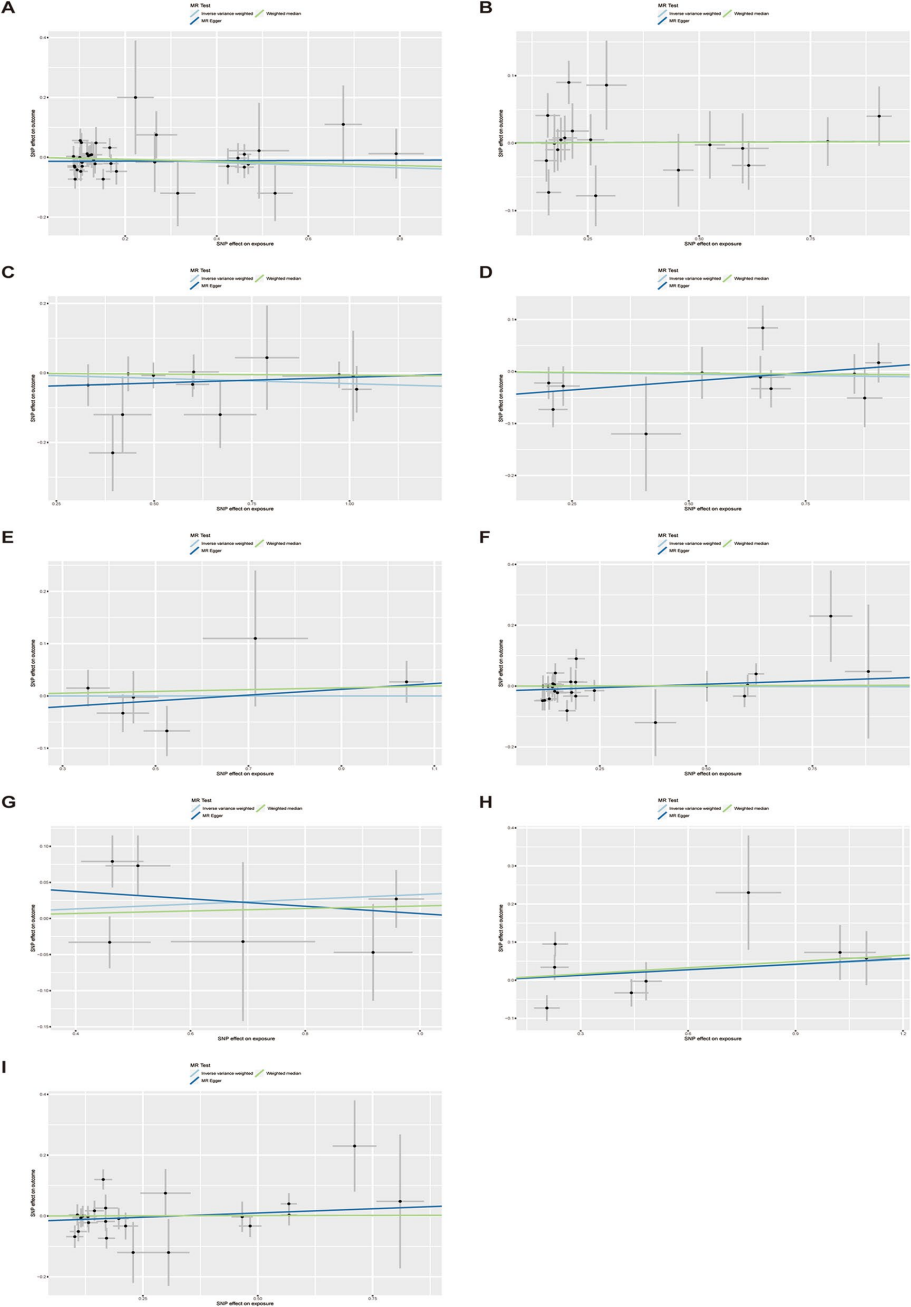
Scatterplots for the significant MR association (*P* < 0.05) between type 1 diabetes and PCOS. **A**, genetically predicted type1 diabetes wide definition on PCOS; **B**, genetically predicted type1 diabetes early onset on PCOS; **C**, genetically predicted type 1 diabetes with coma on PCOS; **D**, genetically predicted type 1 diabetes with ketoacidosis on PCOS; **E**, genetically predicted type 1 diabetes with neurological complications on PCOS; **F**, genetically predicted type 1 diabetes with ophthalmic complications on PCOS; **G**, genetically predicted type 1 diabetes with peripheral circulatory complications on PCOS; **H**, genetically predicted type 1 diabetes with renal complications on PCOS; I, genetically predicted type 1 diabetes with other specified/multiple/unspecified complications on PCOS. SNP, single nucleotide polymorphism

Among the tested type 1 diabetes nine subgroups, we did not observe evidence of causal association between type 1 diabetes and PCOS using the IVW analysis: type1 diabetes wide definition (OR = 0.96, 95% CI 0.90–1.01; *P* = 0.14); type1 diabetes early onset (OR = 1.00, 95% CI 0.95–1.06; *P* = 0.92); type 1 diabetes with coma (OR = 0.97, 95% CI 0.93–1.02; *P* = 0.20); type 1 diabetes with ketoacidosis (OR = 0.99, 95% CI 0.95–1.06; *P* = 0.65); type 1 diabetes with neurological complications (OR = 1.00, 95% CI 0.94–1.06; *P* = 1.00); type 1 diabetes with ophthalmic complications (OR = 1.00, 95% CI 0.94–1.06; *P* = 0.94); type 1 diabetes with peripheral circulatory complications (OR = 1.03, 95% CI 0.96–1.11; *P* = 0.36); type 1 diabetes with renal complications (OR = 1.05, 95% CI 0.94–1.16; *P* = 0.40); and type 1 diabetes with other specified/multiple/unspecified complications (OR = 1.00, 95% CI 0.93–1.08; *P* = 0.95). Meanwhile, similar risk estimates were obtained using the MR-Egger regression and weighted median approaches; though the association was also not statistically significant (Fig. 2). And three MR estimates presented consistent direction and magnitude, supporting the robustness of the causality. The MR estimates indicated that genetically predicted increase in T1D, either at early onset or in the presence of comorbidities, was not significantly associated with PCOS risk (Fig. 3).

### Sensitivity analyses

A series of sensitivity analyses were conducted to assess the robustness of the above results, including Cochran’s *Q* test, MR-Egger intercept test, and MR-PRESSO global test (Table 1). Cochran *Q*-derived *P* values (all > 0.05) indicated that no heterogeneity was detected in the IVW model. Besides, all P values of the MR-Egger intercept tests were > 0.05, suggested that no horizontal pleiotropy existed (Table 1). MR-PRESSO was then used to identify SNPs with potential pleiotropy with a threshold of *P* < 0.05, and no pleiotropy was found to exist (Table 1). Furthermore, no single SNP strongly violated the overall effect of T1D on PCOS in the leave-one-out (LOO) sensitivity analysis (Additional file 1: Fig. S1). Further funnel plot analysis revealed that the graphs were symmetrical, indicating that there were no pleiotropy in the study (Additional file 1: Fig. S2).

**Table 1:** Sensitivity analysis of the causal association between type 1 diabetes and PCOS

Exposure	N	Heterogeneity		Pleiotropy		MR-PRESSO
		*Q* value	*P* value	Intercept	*P* value	*P* value
Type 1 diabetes, wide definition	36	30.17	0.70	− 0.01	0.21	0.73
Type 1 diabetes, early onset	18	22.09	0.18	0.0002	0.99	0.25
Type 1 diabetes with coma	12	7.28	0.78	− 0.05	0.38	0.82
Type 1 diabetes with ketoacidosis	11	12.37	0.26	− 0.05	0.08	0.31
Type 1 diabetes with neurological complications	6	4.15	0.53	− 0.04	0.44	0.53
Type 1 diabetes with ophthalmic complications	25	28.67	0.23	− 0.02	0.24	0.25
Type 1 diabetes with peripheral circulatory complications	6	8.30	0.14	0.06	0.48	0.22
Type 1 diabetes with renal complications	8	17.62	0.01	− 0.002	0.97	0.04
Type 1 diabetes with other specified/multiple/unspecified complications	23	32.30	32.30	− 0.02	0.31	0.06

### Replication and meta‑analysis

**Fig. 4:**
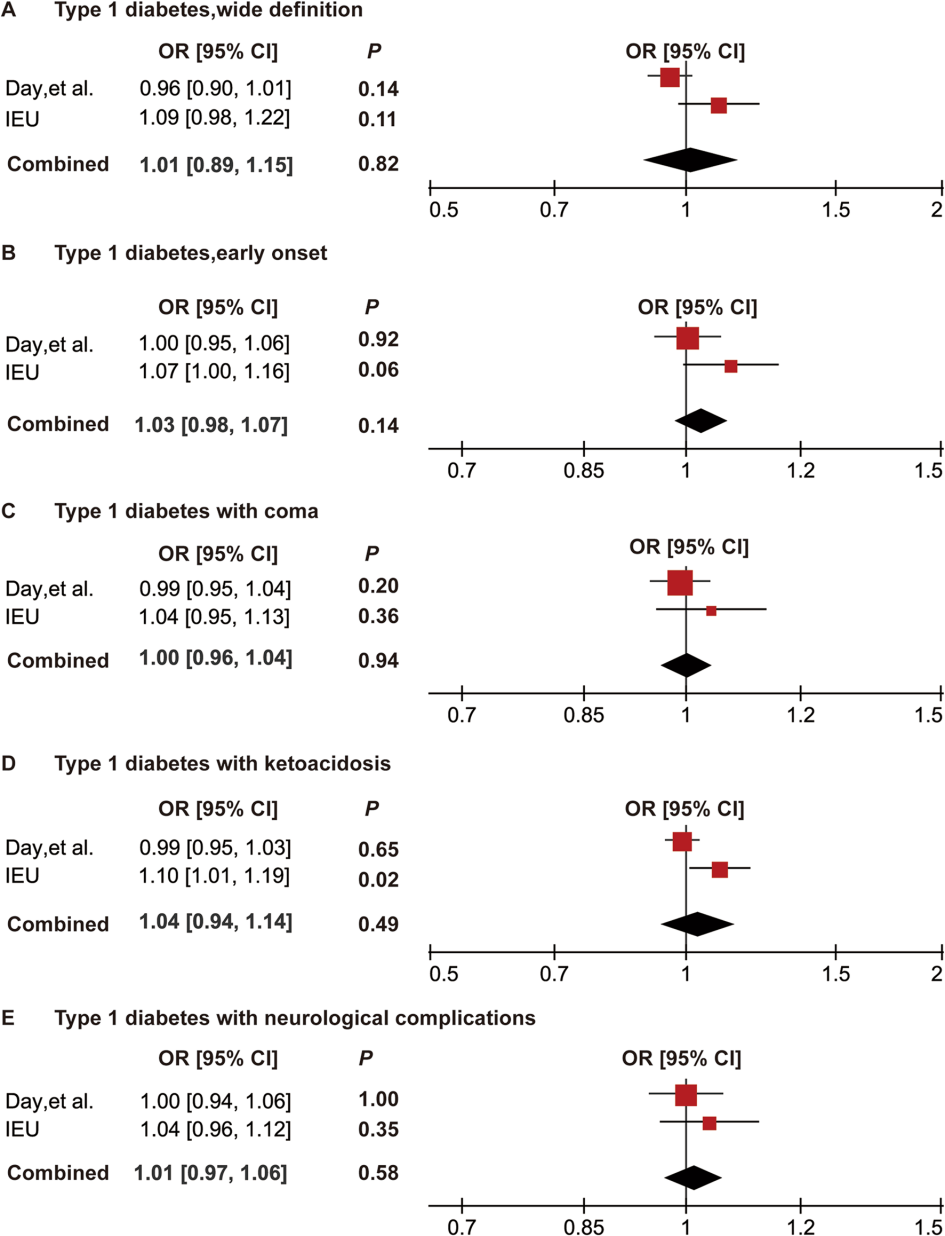
Meta-analysis of the causal associations between type 1 diabetes and PCOS. A**,** Meta-analysis between type1 diabetes wide definition and PCOS; **B**, meta-analysis between type1 diabetes early onset and PCOS; **C**, meta-analysis between type 1 diabetes with coma and PCOS; **D**, meta-analysis between type 1 diabetes with ketoacidosis and PCOS; **E**, meta-analysis between type 1 diabetes with neurological complications and PCOS. OR, odds ratio; CI, confidence interval

**Fig. 5:**
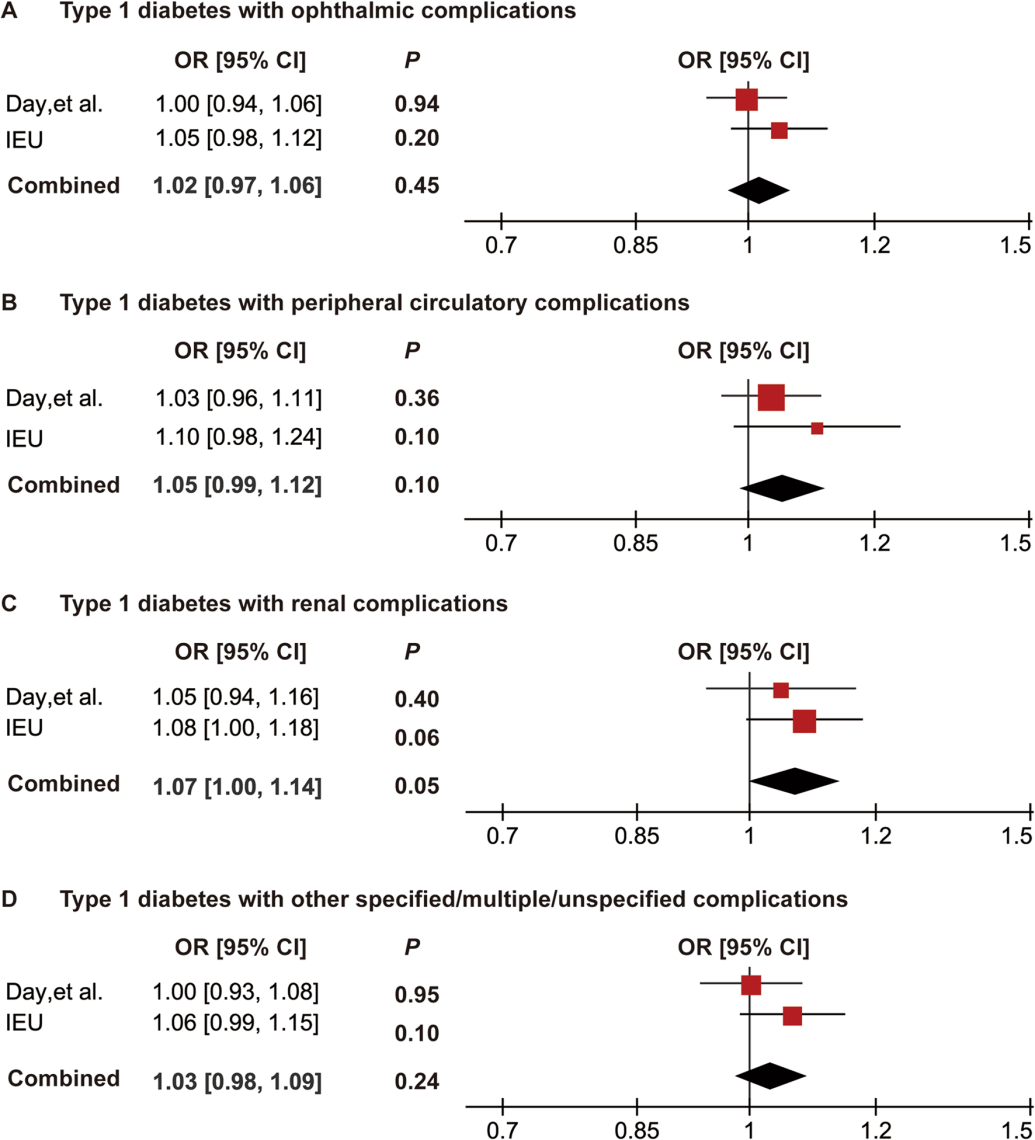
Meta-analysis of the causal associations between type 1 diabetes and PCOS. **A**, Meta-analysis between type 1 diabetes with ophthalmic complications and PCOS; **B**, meta-analysis between type 1 diabetes with peripheral circulatory complications and PCOS;** C**, meta-analysis between type 1 diabetes with renal complications and PCOS; **D**, meta-analysis between type 1 diabetes with other specified/multiple/unspecified complications and PCOS. OR, odds ratio; CI, confidence interval

To further verify our MR results, replication analysis was conducted using PCOS GWAS data from the FinnGen IEU consortium. IVW analysis was performed using the IEU PCOS GWAS data; as expected, similar trends were observed in nine subgroups of type 1 diabetes. P values for all T1D subgroups were greater than 0.05. Meanwhile, the results of the IEU and Day et al. datasets were further combined and meta-analyzed, and all P values were greater than 0.05 with ORs near 1, further determining that there was no causal association between T1D and PCOS. These results further confirm the robustness and credibility of the current MR analysis (Figs. 4 and 5).

### Risk factor analysis

To determine whether the genetically determined MR association between type 1 diabetes and PCOS was disrupted through pleiotropic pathways associated with PCOS, we examined the relationship between T1D and several PCOS risk factors such as body mass index, sex hormone-binding globulin, fasting insulin, waist circumference, hip circumference, and waist-to-hip ratio, using the IVW method. All P values were greater than 0.05, which indicated that no causal effects of T1D on potential PCOS risk factors were observed (Table 2).

**Table 2:** Mendelian randomization estimates of the associations from type 1 diabetes (wide definition) on common risk factors

Outcome	OR (95% CI)	*P* value
BMI (kg/m^2^)	1.00 (0.99, 1.00)	0.53
Sex hormone-binding globulin (SHBG)	1.00 (0.99, 1.01)	0.49
Fasting insulin	1.01 (1.00, 1.01)	0.11
Waist circumference	1.00 (0.99, 1.00)	0.65
Hip circumference	0.99 (0.98, 1.00)	0.16
Waist-to-hip ratio	1.00 (0.99, 1.02)	0.62

## Discussion

We used a two-sample MR approach to comprehensively assess whether T1D causally affects the incidence of PCOS and suggest that T1D per se does not have a causal relationship with PCOS.

In order to make the results more credible and robust, we detailed type 1 diabetes into nine subgroups, including: type1 diabetes wide definition, type1 diabetes early onset, type 1 diabetes with coma, type 1 diabetes with ketoacidosis, type 1 diabetes with neurological complications, type 1 diabetes with ophthalmic complications, type 1 diabetes with peripheral circulatory complications, type 1 diabetes with renal complications, and type 1 diabetes with other specified/multiple/unspecified complications. As far as possible, all the conditions of T1D were considered. And we adopted the strong IVs from the latest and largest GWAS of respective phenotypes in European populations [21, 22]. Meanwhile, we used the GWAS data of PCOS from two databases as the endpoints for the MR analysis and performed meta-analysis of the results at the end. Despite our detailed delineation of T1D and validation of different database replications, no causal relationship was found between each subgroup and PCOS.

It is widely recognized that the development of T1D and PCOS is closely related to intrinsic genetic factors [23, 24]. T1D, as an autoimmune disease in which the patient's own immune system destroys insulin-secreting β-cells in the pancreas, and genetic factors play an important role in the onset, progression, and eventual clinical diagnosis of type 1 diabetes [25]. Genes in the human leukocyte antigen (HLA) region are the primary susceptibility genes for T1D, and non-HLA genes (INS, CTLA4, PTPN22 genes, etc.) also play a role in the pathogenesis of T1D [24]. A GWAS in a Chinese Han population reported five independent susceptibility loci strongly associated with T1D, namely rs4320356 near the BTN3A1 gene, rs3802604 in the GATA3 gene, rs1770 in the MHC gene, rs705699 in the SUOX gene, and HLA-C 275 [26]. Patients with PCOS often exhibit hyperactivation of the hypothalamic–pituitary–adrenal (HPA) axis, and some studies have confirmed the involvement of the KiSS1 and GNRH genes on the HPA axis in the development of PCOS [27, 28]. Meanwhile, recent researchers detected 22 corticotropin-releasing hormone receptor (CRHR) CRHR1 variants and 1 CRHR2 variant significantly associated with PCOS (*P* < 0.05) in different genetic models [29]. Five new variants in the dopamine receptor 2 (DRD2) gene that are significantly associated with PCOS risk were also recently identified [30]. Not only that, but CRHR and DRD2 gene variants are likewise correlated with the development of diabetes mellitus [29, 30]. Therefore, it would be interesting to explore whether there is a correlation or causal relationship between T1D and PCOS in terms of genetic markers.

To date, it is inconclusive whether there is a causal effect of T1D on the development of PCOS [17, 31–33]. In a recent meta-analysis that included 19 studies analyzing the prevalence of T1D and PCOS, it was found that pooled prevalence of classic PCOS (US National Institutes of Health criteria) in T1D was 16% (95% CI: 10–22%) [31]. However, the heterogeneity was high in almost all these meta-analyses (*P* < 0.001), with an estimated predictive interval of 8–59%. At the same time, more than 89% of the studies included in the meta-analysis were cross-sectional and case–control studies, with a vague chronological order of the onset of the two diseases, and a large number of confounders and biases, which did not allow for a clear causal relationship [31]. And about 1/4 of the included studies only described hyperandrogenic traits and did not meet any of the valid definitions for PCOS [31]. On the contrary, the advantage of two-sample Mendelian randomization can effectively circumvent precisely these shortcomings of observational studies, which may be one of the possible reasons for the inconsistency between the results of our MR study and those of previous observational studies. As indicated by Mendel's laws of inheritance, alleles segregate randomly from parents to offspring [34]. Thus, MR effectively avoids the effects of multiple population confounders. In addition, germline genotypes are fixed at conception and precede the observed variables, effectively avoiding the problem of reverse causality [34]. At the same time, the genotypes studied by MR are ethnographic category concepts [34], which excludes the influence of factors such as insufficient sample size, and can better represent the whole and obtain more reliable conclusions.

In addition, in response to the previously described reasons for the inconsistency between the observational study and our MR results, a possible explanation is a key consideration—the age of the study participants. Because PCOS is a condition that primarily affects women of productive age, the majority of observational studies have been conducted in predominantly young women. Whereas the onset of T1D commonly occurs in childhood or adolescence, it can also occur at all ages throughout life, with menopause also being a high-risk period [35, 36]. Though we considered the effects of early-onset T1D in the current MR analysis, the diagnosis of PCOS, on the other hand, lacks diagnostic criteria in adolescence. Moreover, as PCOS patients with age, the syndrome may improve or even subside completely, with a decrease in both ovarian size and androgen production [37]. The existence of these problems may ultimately influence our conclusions on causal associations. Future large-scale prospective studies in women with detailed subgroups of T1D according to age, categorized as prepubertal to menopause and beyond, with detailed descriptions of each group, are needed to obtain more reliable results.

Another possible explanation for our negative results is that the currently used GWAS data for PCOS may employ multiple heterogeneous diagnostic criteria and may be a collection of multiple different pathophysiologic outcomes. If some of these are causally associated with T1D, but when combined, they may reduce the ability to detect a genetic association between T1D and PCOS. The largest European PCOS GWAS, which served as our outcome data, is liberal in its diagnostic criteria for PCOS, allowing not only the NIH (14.6% of included cases) and Rotterdam criteria (34.0%), but also self-reported diagnoses (51.4%) [22]. The accuracy of these diagnostic criteria may vary, which ultimately may have affected the accuracy of the results of the MR analysis and the specificity. Future testing of large samples of GWAS for different PCOS subtypes may contribute to better MR work on PCOS and other diseases and clearer causality [38].

Although the analysis of our study in terms of genetic factors suggests that T1D does not cause PCOS, the causal relationship between T1D and PCOS may depend on more than just genetic factors, and several of the common features of T1D do seem to contribute to PCOS, which may also explain the epidemiologic association. Hyperandrogenemia is one such feature [39]. Subcutaneous injections of supraphysiologic doses of insulin used in the routine treatment of patients with T1D will result in exogenous systemic hyperinsulinemia. Hyperinsulinemia not only increases the secretion of gonadotropins and adrenocorticotropic hormone, but also increase androgen secretion by co-stimulating the production of ovarian and adrenal hormones, and hyperandrogenemia occurs [39]; hyperandrogenism in turn further leads to visceral/abdominal fat deposition, aggravating insulin resistance and hyperinsulinemia [40]. Hyperandrogenemia as one of the common clinical manifestations of PCOS is likely to induce PCOS.

Not only that, but another important characteristic of patients with T1D may also have an impact on the development of PCOS. It has been found that T1D women have increased levels of growth hormone (GH) and decreased levels of insulin-like growth factor 1 (IGF1), which is especially common in T1D patients with poor glycemic control [41, 42]. Expression of hepatic GH receptors is partly dependent on insulin, and in T1D patients, because of the decrease in insulin exported from the portal vein, the stimulation of hepatic synthesis and secretion of IGF1 is also reduced, which leads to negative feedback, and the secretion of GH is continuous, and this alteration of the GH/IGF1 axis also contributes to the development and progression of insulin resistance in patients with T1D. IGF1 axis is over-activated, which further promotes high insulin levels in the blood of T1D patients to over-stimulate insulin receptors and IGF1 receptors in the ovaries, increase the synthesis and secretion of steroid hormones, and promote the development of PCOS [43].

At the same time, immune factors may be involved in causally influencing the development of PCOS disease in patients with T1D. Ovarian tissue serves as a common target of attack in a variety of autoimmune diseases, and a variety of humoral and cytokines involved in the autoimmune response may affect ovarian function [44]. Studies have found an increased risk of PCOS in patients with autoimmune thyroid disease, primary chronic hypoadrenocorticism (i.e., Addison's disease), rheumatoid arthritis, and systemic lupus erythematosus [45, 46]. Type 1 diabetes, as an autoimmune disease [8], autoimmune antibodies may cause a cytotoxic reaction during follicular development and attack steroid-producing cells during follicular maturation through the follicle-blood barrier, resulting in autoimmune ovarian inflammation, which leads to the normalization or enlargement of the size of the involved ovaries, and thus contributes to the development of PCOS [47]. Therefore, despite the negative results of our MR study, the causal relationship between T1D and PCOS and the associated clinical significance should be approached with caution, and future clinical data with larger sample sizes as well as more in-depth studies are needed for validation.

The current study has several strengths. First, we performed the first state-of-the-art two-sample MR analysis of T1D and PCOS using robust GWAS loci. Unlike traditional observational studies, our analysis represents a large number of individuals with T1D for inferring causal associations for PCOS. Meanwhile, we divided T1D into nine subgroups and analyzed them separately, which is the most comprehensive and systematic study on T1D to date. Second, using the MR design, our study is largely free of reverse causality and confounders. Specifically, MR results were calculated using a range of methods to ensure the reliability of the MR analysis. The robustness of the MR estimates was demonstrated by using different MR analysis models that yielded results of consistent direction and similar magnitude. And the robustness of our results is supported by the sensitivity analyses. Third, replication and meta-analysis were applied to further clarify the causal effect of T1D on PCOS. The final effect estimates were consistent regardless of which consortium's data were used. These consistent results are reassuring because they do not appear to have occurred by chance. Further meta-analysis showed that T1D still had no effect on PCOS.

There are some limitations should be noted in our study. First, the majority of participants in this study were European. Although this largely avoids the influence of population heterogeneity on the results, applying the results of the MR analysis to other ethnic groups requires more caution. Therefore, future validation of GWAS data from more ethnographic sources is needed for better generalization. Second, the relatively low proportion of PCOS cases in the database may have resulted in reduced statistical power to detect true causality. Therefore, we performed repeated analyses using two independent datasets to validate the robustness of the MR estimates, thus greatly improving the confidence of our results. Finally, although the MR approach excels in causal reasoning, we should be wary that the results of this MR study should be further validated in robust randomized controlled trials to demonstrate a causal relationship.

If further validation is performed in future with more SNPs and larger cohorts, the current results will have important implications for how clinicians manage patients with T1D, especially regarding the risk of PCOS in women. Although the current study does not support T1D per se as an indication for a PCOS prevention strategy, other studies have emphasized the importance of hyperandrogenemia, increased LH/FSH ratios, high levels of 17-hydroxyprogesterone women are at increased risk of developing PCOS [17, 32, 33]. Taken together, these studies suggest that women with T1D with these characteristics are best suited to focus on prevention of PCOS, and we will be better able to provide risk counseling if it is supported by large prospective studies in future. Considering that T1D per se does not increase the risk of developing PCOS, women with T1D who are of normal weight and have normal androgen levels do not need to experience the stress of being told they are at increased risk of developing PCOS; however, they should be counseled to avoid weight gain, which may increase the risk of developing the disease [48]. This study is an example of how genetic data can lead to personalized medicine.

## Conclusion

In conclusion, this is the first MR study to investigate the causal relationship between type 1 diabetes and PCOS. Our MR analysis does not support the hypothesis that type 1 diabetes could increase the incidence of PCOS. Given these result, prevention of PCOS complications in women with T1D should focus on women with high-risk features rather than all women with T1D. There is a strong need for larger MR studies and RCTs in future to confirm the results of this study.

## Methods

### Study design

In this study, the two-sample Mendelian randomization analysis was implemented to examine the causal effects of type 1 diabetes (including multiple comorbidity subgroups) on polycystic ovarian syndrome using GWAS summary statistics. For a valid interpretation for the MR analysis, the following three fundamental assumptions must fulfill: (1) genetic instruments are robustly correlated with the exposure; (2) genetic instruments are independent of any confounders between the relationship of exposure and outcome; and (3) genetic instruments influence the outcome only via exposures of interest [49]. The second and third of these assumptions, collectively referred to as the independence of horizontal pleiotropy, can be tested using a range of statistical methods [50]. Genetic information on PCOS was obtained from two independent GWAS consortia for primary and replication analysis, followed by a meta-analysis.

The flowchart of the current MR design is presented in Fig. 1. Our MR studies were conducted using publicly published studies or shared datasets that had received ethical approval and informed consent. No additional ethical statements or consents were required.

### GWAS data for type 1 diabetes

The GWAS summary statistics data of type 1 diabetes were obtained from the latest release from the FinnGen consortium in January 2023 [21]. According to the definition criteria of type 1 diabetes and different comorbidities, the T1D was analyzed in an exhaustive and detailed manner by taking into account the various conditions of T1D, divided into nine groups. Specifically, this includes a broad definition of T1D, early-onset T1D at younger than 20 years of age, T1D combined with acute complications of diabetic ketoacidosis, T1D combined with acute complications of advanced neuropsychiatric symptoms-coma, and T1D combined with a variety of chronic complications: neuropathy, ophthalmic complications, peripheral circulatory complications, diabetic nephropathy, and other specific, multiple, or nonspecific complications. The nine groups are listed as follows: type1 diabetes, wide definition (8967 cases and 308,373 controls); type1 diabetes, early onset (2770 cases and 374,507 controls); type 1 diabetes with coma (2050 cases and 308,280 controls); type 1 diabetes with ketoacidosis (2102 cases and 308,280 controls); type 1 diabetes with neurological complications (1077 cases and 308,280 controls); type 1 diabetes with ophthalmic complications (5202 cases and 308,280 controls); type 1 diabetes with peripheral circulatory complications (669 cases and 308,280 controls); type 1 diabetes with renal complications (1579 cases and 308,280 controls); type 1 diabetes with other specified/multiple/unspecified complications (6234 cases and 308,280 controls), adjusted for sex, age, genotyping batch, and the first 10 genetic principal components. The detailed information of each GWAS summary statistic in this study is listed in Additional file 2: Table S1. And Information on International Classification of Diseases (ICD) codes that were used to define cases of T1D and PCOS is presented in Additional file 2: Table S2.

### GWAS data for PCOS

The GWAS data of PCOS were obtained from the latest and largest published GWAS meta-analysis conducted by Day et al., including 10,074 patients with PCOS and 103,164 health controls of European ancestry, and were adjusted for age [22, 24]. And diagnosis of PCOS was according to the National Institutes of Health (NIH) criteria [25, 51], Rotterdam criteria [6], or self-reported questionnaire [52]. More details on demographics and quality control were described in the initial GWAS study. To validate our results through replication analysis and meta-analysis, we used the PCOS data from IEU consortium (642 PCOS cases and 118,228 healthy controls), which can be publicly available on the website at https://gwas.mrcieu.ac.uk. The detailed information of each GWAS summary statistic in this study is listed in Additional file 2: Table S1.

### Instruments selection

Eligible genetic variants associated with T1D are selected through a series of steps. Screening was determined according to the criteria proposed by Martin Bahls et al. [53]. Firstly, SNPs were associated with the exposure at the genome-wide significance levels (*P* < 5 × 10^−8^). In addition, we aggregated SNPs in linkage disequilibrium using the PLINK algorithm (LD, *R*^2^ threshold = 0.001 and window size = 10 mb). Meanwhile, to avoid bias caused by the use of weak instruments, the F-statistic of SNPs was calculated to measure statistical strength, as described earlier [54]. SNPs with *F* < 10 were excluded to ensure that all the SNPs were strongly associated with exposure when used as IVs [20]. We then extracted the exposure SNPs from the outcome data and excluded associated with the outcome (*P* < 5 × 10^−8^). Harmonizing processes were then conducted to align the alleles of exposure- and outcome-SNPs, and discard palindromic SNPs with intermediate effect allele frequencies (EAF > 0.42) or SNPs with ambiguous alleles. Finally, SNPs with potential pleiotropy were removed after MR-pleiotropy residual sum and outlier (MR-PRESSO), and MR analysis was reperformed to evaluate the robustness. After a series of rigorous filtering steps above, 36, 18, 12, 11, 6, 25, 6, 8 and 23 SNPs were used as IVs for type1 diabetes wide definition; type1 diabetes early onset; type 1 diabetes with coma; type 1 diabetes with ketoacidosis; type 1 diabetes with neurological complications; type 1 diabetes with ophthalmic complications; type 1 diabetes with peripheral circulatory complications; type 1 diabetes with renal complications and type 1 diabetes with other specified/multiple/unspecified complications in our study, respectively (Additional file 2: Tables S3–S11).

### Mendelian randomization analyses

Four MR analytical methods were applied to evaluate the causal effects of T1D and PCOS in this study. The random-effects inverse-variance weighted (IVW) method was conducted as the primary analysis. IVW combines all the Wald ratio obtained from each of the SNPs and used a meta-analytic approach to derive the causal effect of exposure on the outcome. At the same time, the random-effects IVW method obtains a more conservative and plausible causal inference accounting for the pleiotropy [55]. Unless otherwise specified, all "IVW" herein refers to random-effects IVW. We considered a statistically significant results were identified at *P* < 0.05 threshold. Furthermore, additional MR analyses, such as MR-Egger regression, weighted median, and Mendelian randomization pleiotropy residual sum and outlier (MR-PRESSO) methods, were implemented as complements to the IVW, because these methods could provide more reliable estimates under a wider range of scenarios. The weighted median assumes that at least half of the instruments are valid [56]. MR-Egger is the lowest power method, but still provides consistent estimates when all the instruments are invalid, taking into accounting for pleiotropy [57]. Moreover, if results from different MR analyses were inconsistent, recalculated and use more stringent instrumental P-value thresholds [58].

### Sensitivity analysis

Sensitivity analysis was then conducted to access the robustness of the MR analyses. We detected heterogeneity using the Cochran Q-test [59]. We considered that there was potential heterogeneity when *P* < 0.05 and *I*^2^ > 25% in the Cochran *Q*-test. Meanwhile, MR-Egger intercept test and MR-PRESSO global test were performed (Table 1). All *P* values of Cochran *Q*-test were > 0.05, indicating that no heterogeneity existed (Table 1). And Egger intercepts did not detect any pleiotropy, indicating that the MR estimates did not introduce a pleiotropic bias. Other analyses did not reveal heterogeneity. Leave-one-out (LOO) analysis was conducted to evaluate whether any single SNP drove the results. And the presence of outliers can be detected based on whether the funnel plot is symmetric or not.

### Replication and meta‑analysis

To validate the robustness of the MR analysis, we replicated IVW analysis using another independent PCOS GWAS data from the IEU consortium mentioned above and then conducted a meta-analysis to determine the final results.

### Risk factors

To further investigate potential mediators between type 1 diabetes and increased risk of polycystic ovary syndrome, we assessed the effect of type 1 diabetes on several common risk factors for PCOS using MR methods. As shown in Table 2, body mass index (BMI), sex hormone-binding globulin (SHBG), fasting insulin, waist circumference (WC), hip circumference (HC), and waist-to-hip ratio (WHR) were selected as potential risk factors. Insulin resistance was represented by fasting insulin. The GWAS summary statistics of BMI, SHBG, WC, HC, WHR, and fasting insulin were all from the publicly available IEU Open GWAS Project database (https://gwas.mrcieu.ac.uk/), for which details are shown in Additional file 2: Table S1.

### Statistical analysis

Statistical significance was set at *P* < 0.05. And the MR estimates, expressed as odds ratios (OR) and corresponding 95% confidence intervals (CI), provide an estimate of the relative risk of PCOS for each standard deviation (SD) increase in type 1 diabetes. All statistical analyses were performed by the two-sample MR package (version 0.5.6) and MR-PRESSO package (version 1.0) of the R program (version 4.1.2).

### Supplementary Information

**Additional file 1: Figure S1** The leave-one-out analysis plot. Figure S2 Funnel plots from genetically predicted type 1 diabetes on PCOS.

**Additional file 2: Table S1** Description of GWAS summary samples used in this study. **Table S2** The International Classification of Diseases (ICD) code was used to define cases of T1D and PCOS. **Table S3** Instrument variables of type1 diabetes, wide definition. **Table S4** Instrument variables of type1 diabetes, early onset. **Table S5** Instrument variables of type1 diabetes with coma. **Table S6** Instrument variables of type1 diabetes with ketoacidosis. **Table S7** Instrument variables of type1 diabetes with neurological complications. **Table S8** Instrument variables of type1 diabetes with ophthalmic complications. **Table S9** Instrument variables of type1 diabetes with peripheral circulatory complications. **Table S10** Instrument variables of type1 diabetes with renal complications. **Table S11** Instrument variables of type1 diabetes with other specified/multiple/unspecified complications.

## Data Availability

All data generated or during this study are included in this published article and the supplementary materials.

## Abbreviations

MR: Mendelian randomization
T1D: Type 1 diabetes
PCOS: Polycystic ovary syndrome
RCTs: Randomized controlled trials
SNPs: Single nucleotide polymorphisms
IVs: Instrumental variables
GWAS: Genome-wide association study
IVW: Inverse-variance weighted
WM: Weighted median
MR-PRESSO: MR-pleiotropy residual sum and outlier
LOO: Leave-one-out
BMI: Body mass index
SHBG: Sex hormone-binding globulin
WC: Waist circumference
HC: Hip circumference
WHR: Waist-to-hip ratio
OR: Odds ratios
CI: Confidence intervals
