# Supplementary material for: Genetic evidence for the causal association between type 1 diabetes and the risk of polycystic ovary syndrome
Source: Hum Genomics. 2023 Nov 13;17:100. doi: 10.1186/s40246-023-00550-z (PMC10641977; doi:10.1186/s40246-023-00550-z)
Supplement: Supplementary file 1 — Additional file 1: Figure S1 The leave-one-out analysis plot. Figure S2 Funnel plots from genetically predicted type 1 diabetes on PCOS. [file 40246_2023_550_MOESM1_ESM.docx]

Supplementary Material

# Supplementary Figures


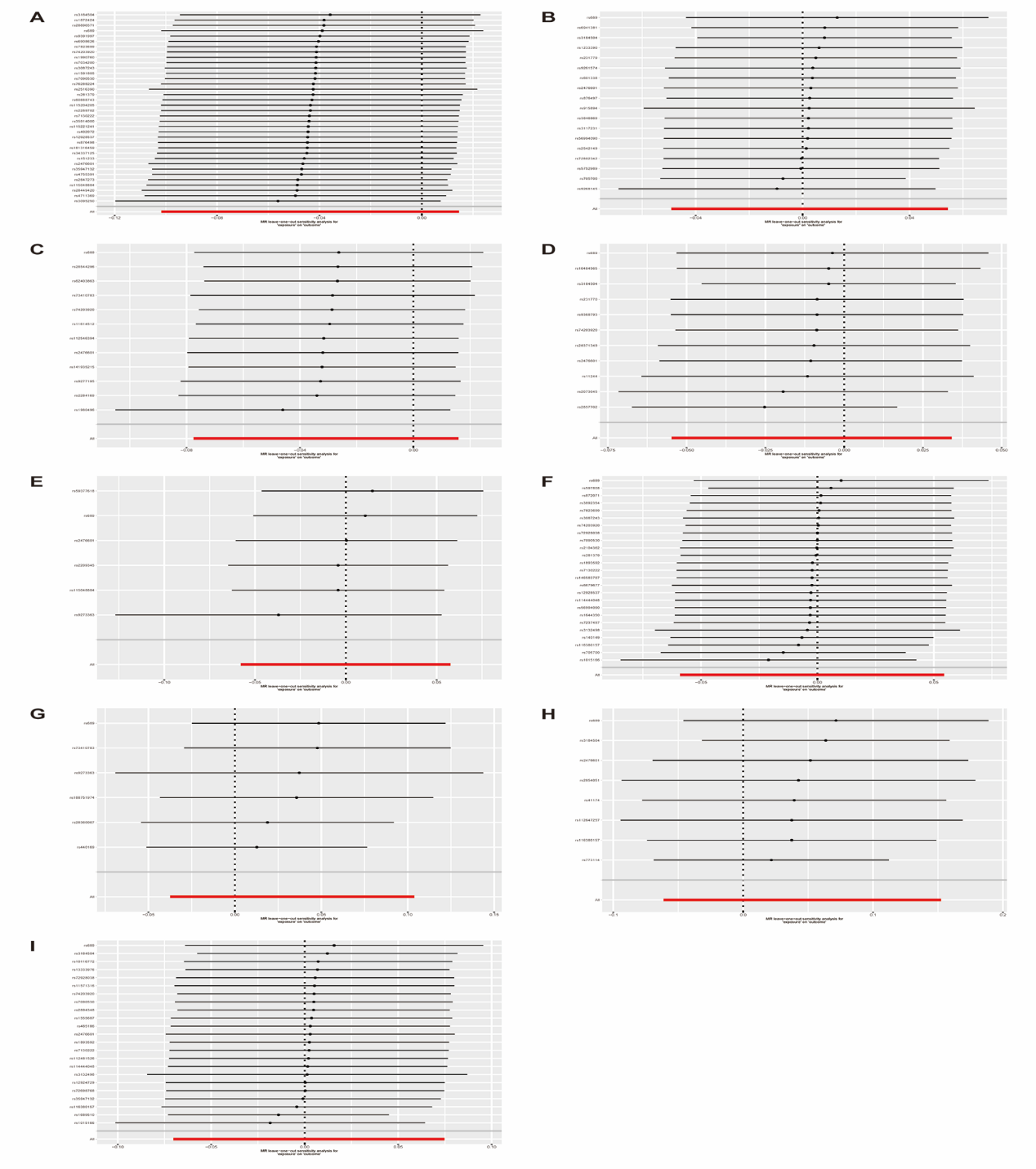


**Supplementary Figure 1.** The figure legends are required to have the same font as the main text, 12 point normal Times New Roman, single spaced. Please use a single paragraph for each legend and prepare the figures keeping in mind the PDF layout.


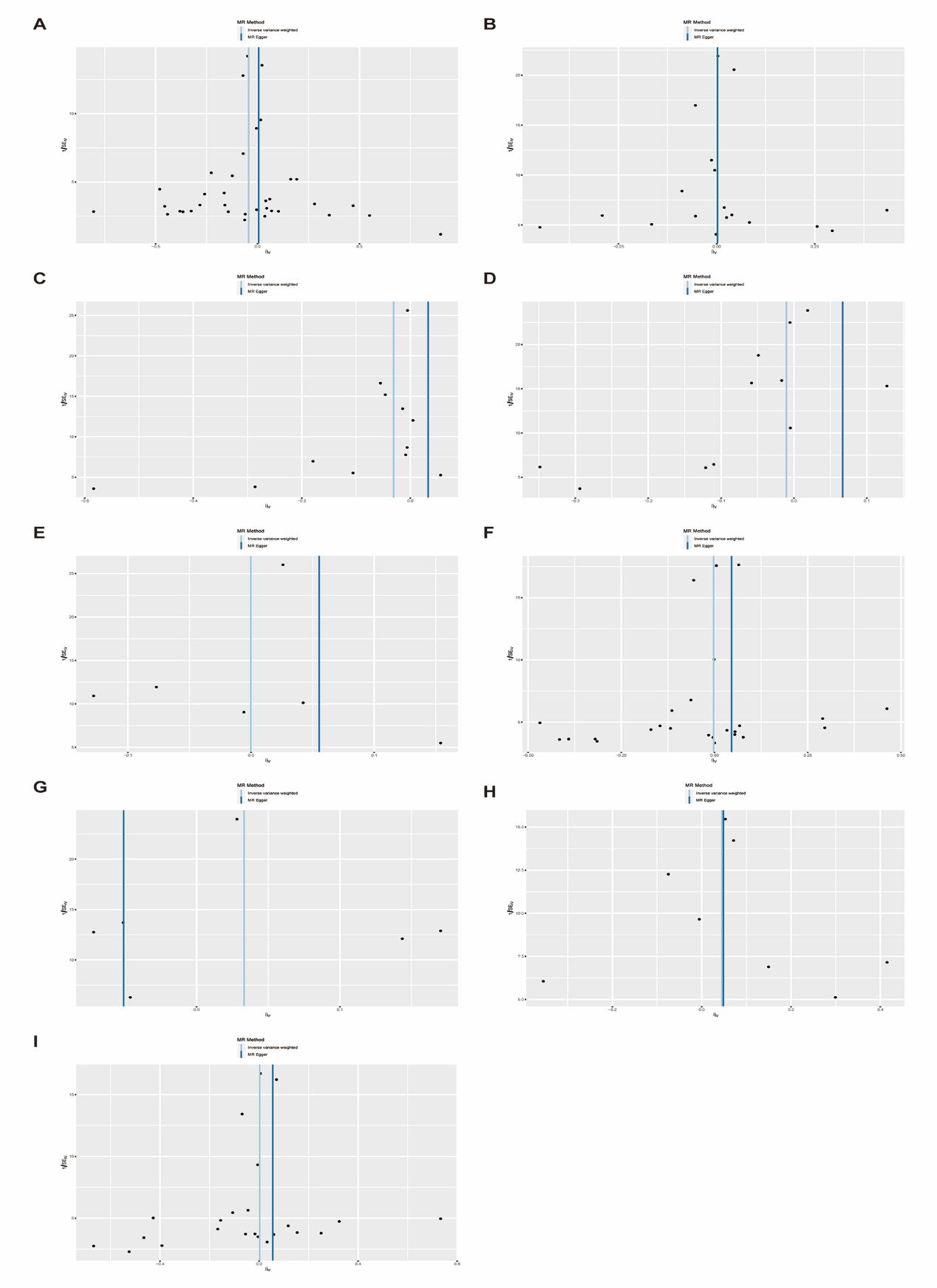


**Supplementary Figure 2.** Funnel plots from genetically predicted type 1 diabetes on PCOS. A, Funnel plot for type1 diabetes wide definition on PCOS; B, Funnel plot for type1 diabetes early onset on PCOS; C, Funnel plot for type 1 diabetes with coma on PCOS; D, Funnel plot for type 1 diabetes with ketoacidosis on PCOS; E, Funnel plot for type 1 diabetes with neurological complications on PCOS; F, Funnel plot for type 1 diabetes with ophthalmic complications on PCOS; G, Funnel for type 1 diabetes with peripheral circulatory complications on PCOS; H, Funnel plot for type 1 diabetes with renal complications on PCOS; I, Funnel plot for type 1 diabetes with other specified/multiple/unspecified complications on PCOS. SNP, single nucleotide polymorphism.
